# Sleep Deficit and Inflammatory Markers as Combined Risk Factors for Glaucoma Progression: A Prospective Longitudinal Observational Cohort Study

**DOI:** 10.3390/ijms27031338

**Published:** 2026-01-29

**Authors:** Raluca Neacşa, Cristiana Tănase, Adina-Diana Moldovan, Daniela Manasia, Mădălina-Elena Tobă

**Affiliations:** 1 Medical Doctoral School, Titu Maiorescu University of Bucharest, 040317 Bucharest, Romania; raluca.neacsa@prof.utm.ro; 2 Department of Ophthalmology, Witting Clinical Hospital, 010243 Bucharest, Romania; danamanasia@yahoo.com; 3 Department of Preclinical Disciplines, Faculty of Medicine, Titu Maiorescu University of Bucharest, 031593 Bucharest, Romania; bioch@vbabes.ro; 4 ”Victor Babeș” National Institute of Pathology, 050096 Bucharest, Romania; 5 MedLife SA, 010719 Bucharest, Romania; 6 Department of Medico-Surgical Disciplines, Faculty of Medicine, Titu Maiorescu University of Bucharest, 031593 Bucharest, Romania; madalina.toba@prof.utm.ro; 7 Department of General Surgery, Witting Clinical Hospital, 010243 Bucharest, Romania

**Keywords:** glaucoma, sleep deficit, inflammation, IL-6, TNF-α, neurodegeneration, risk stratification

## Abstract

Glaucoma progression differs markedly between individuals despite comparable intraocular pressure control, implying additional modifiable contributors to neurodegeneration. We evaluated the joint impact of sleep deficit and inflammatory cytokine trajectories on retinal nerve fiber layer (RNFL) loss. In this 24-month prospective longitudinal observational cohort, 57 participants (19 controls, 19 prostaglandin-treated glaucoma, 19 untreated glaucoma) underwent spectral-domain OCT, validated sleep assessment, and serial IL-6 and TNF-α profiling. Longitudinal models tested independent and interactive effects of sleep deficit and inflammation on RNFL change, and mediation analyses assessed whether inflammation explains the sleep–progression association. RNFL loss rates were −0.20 ± 0.10 μm/year (controls), −1.06 ± 0.89 μm/year (treated), and −1.94 ± 0.78 μm/year (untreated; *p* < 0.001). Sleep deficit correlated with RNFL loss in glaucoma (r = −0.41, *p* = 0.010) but not controls, with stronger effects in untreated disease (*p* = 0.034). Each hour of sleep deficit was associated with 0.09–0.11 μm/year faster RNFL loss (*p* < 0.05). A combined sleep–inflammation model improved risk stratification (C-statistic = 0.68). Mediation was not supported. Sleep deficit and inflammatory cytokines act as parallel, independent risk factors for glaucoma progression. Integrating sleep and inflammatory profiling may enhance personalized risk assessment beyond pressure-based management.

## 1. Introduction

Glaucoma affects over 70 million people worldwide and remains the leading cause of irreversible blindness despite advances in intraocular pressure (IOP) management [1]. The substantial heterogeneity in progression rates among patients with similar IOP control suggests that additional biological pathways modulate neurodegeneration beyond mechanical factors alone. Among candidate pathways, sleep-dependent processes and neuroinflammation have emerged as particularly promising given their established roles in neural health and their potential modifiability.

Emerging evidence implicates sleep disturbance and neuroinflammation as independent risk factors for neurodegenerative diseases [2]. In glaucoma, recent studies suggest associations between sleep disorders and disease prevalence [3], while inflammatory cytokines correlate with structural damage [4,5,6]. However, the integrated effects of sleep and inflammation on glaucoma progression remain unexplored, limiting our ability to identify and manage high-risk patients.

Mechanistically, sleep deprivation is associated with inflammatory activation through NF-κB pathways (a master regulator of inflammatory gene expression) [7], and elevated interleukin-6 (IL-6) and tumor necrosis factor-α (TNF-α) [8]. These cytokines promote retinal ganglion cell apoptosis [9] and compromise blood–retinal barrier integrity [10,11]. Additionally, the glymphatic system (the brain’s waste-clearance pathway, most active during sleep) is hypothesized to facilitate clearance of neurotoxic proteins [12,13], with disruption potentially accelerating neurodegeneration. Conversely, glaucomatous damage may disrupt circadian regulation through loss of intrinsically photosensitive retinal ganglion cells [14], creating potential bidirectional relationships.

Previous studies have typically examined sleep or inflammation in isolation, using cross-sectional designs that cannot capture temporal dynamics. Furthermore, dividing patients into simple categories (e.g., “good sleeper” vs. “poor sleeper”) may obscure the gradual relationship between sleep duration and disease risk [15]. To address these limitations, we conducted a prospective longitudinal observational cohort study examining the integrated effects of sleep deficit and inflammatory cytokine trajectories on RNFL progression over 24 months.

Our primary hypothesis was that sleep deficit accelerates RNFL loss in glaucoma patients. Our secondary hypotheses were that: inflammatory cytokines would correlate with faster progression, cytokines would partially mediate sleep effects on neurodegeneration, and combined assessment of sleep and inflammation would improve risk stratification beyond traditional clinical factors. Our findings may improve the understanding of how sleep and inflammation independently contribute to glaucoma progression, informing patient counseling regarding the importance of adequate sleep.

## 2. Results

The 57 participants had a mean age of 65.6 ± 7.3 years with balanced sex distribution (Table 1). Groups were well-matched for demographics and comorbidities (all *p* > 0.4). Baseline RNFL thickness differed as expected: for controls, 95.5 ± 6.4 μm; for latanoprost-treated glaucoma, 72.4 ± 10.2 μm; and for untreated glaucoma, 69.7 ± 10.4 μm (*p* < 0.001). Sleep deficit averaged 1.5 ± 1.1 h across all groups without significant baseline differences (*p* = 0.86).

Baseline cytokines showed disease-related elevation: IL-6 was 1.7 ± 1.2 pg/mL in controls, 2.2 ± 2.1 pg/mL in latanoprost-treated, and 3.0 ± 2.6 pg/mL in untreated glaucoma (*p* = 0.185). TNF-α followed similar patterns (0.7 ± 0.5, 0.9 ± 0.9, 1.5 ± 0.9 pg/mL, respectively; *p* = 0.015). All participants completed a 24-month follow-up with >95% visit attendance.

**Figure 1:**
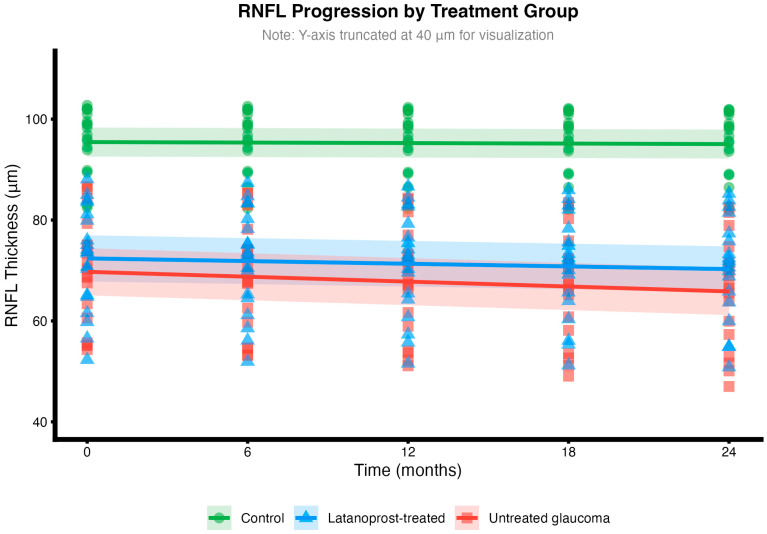
RNFL progression trajectories by group over 24 months. Lines represent mean trajectories with 95% confidence bands. Controls show physiological age-related loss, while glaucoma groups demonstrate accelerated progression, with latanoprost treatment providing partial protection.

Mean RNFL slopes were −0.20 ± 0.10 μm/year in controls (slower than typical age-related loss of 0.5–0.7 μm/year, possibly reflecting our younger cohort or measurement variability), −1.06 ± 0.89 μm/year in latanoprost-treated glaucoma, and −1.94 ± 0.78 μm/year in untreated glaucoma (*p* < 0.001 between groups; Figure 1). Latanoprost treatment was associated with a 0.88 μm/year slower progression (95% CI: 1.43–0.33, *p* = 0.003), representing a 45.4% reduction compared to untreated patients.

Baseline and follow-up IOP control are summarized in Table 2. Mean follow-up IOP remained stable within groups, while between-group differences persisted in line with treatment status.

In pooled glaucoma patients (n = 38), patients with greater sleep deficits had faster RNFL loss (moderate correlation, r = −0.41, 95% CI: −0.65 to −0.11, *p* = 0.0101), explaining approximately 17% of progression variance—meaning that sleep accounts for a meaningful portion of why patients progress at different rates (Figure 1). Controls showed a significant positive correlation (r = 0.47, *p* = 0.04)—interestingly, the opposite direction to glaucoma. This differential effect supports the three-way interaction finding (*p* = 0.034) that sleep effects on progression depend fundamentally on disease status.

The mixed-effects model revealed that sleep deficit effects on RNFL progression differed significantly by treatment group (*p* = 0.034 for this differential effect). In controls, the effect of sleep on progression over time was minimal (coefficient = 0.037, *p* = 0.347), indicating that poor sleep is not associated with faster normal age-related loss. However, in glaucoma patients, sleep deficit was associated with substantially faster progression, particularly in untreated patients, where each hour of deficit was associated with 0.11 μm/year additional RNFL loss. A 2 h sleep deficit in untreated glaucoma would thus be associated with 0.22 μm/year additional loss. In clinical terms, poor sleep is associated with faster nerve fiber loss, particularly in patients whose inflammation is also elevated. These associations, if validated in larger cohorts, could inform individualized monitoring strategies.

**Figure 2:**
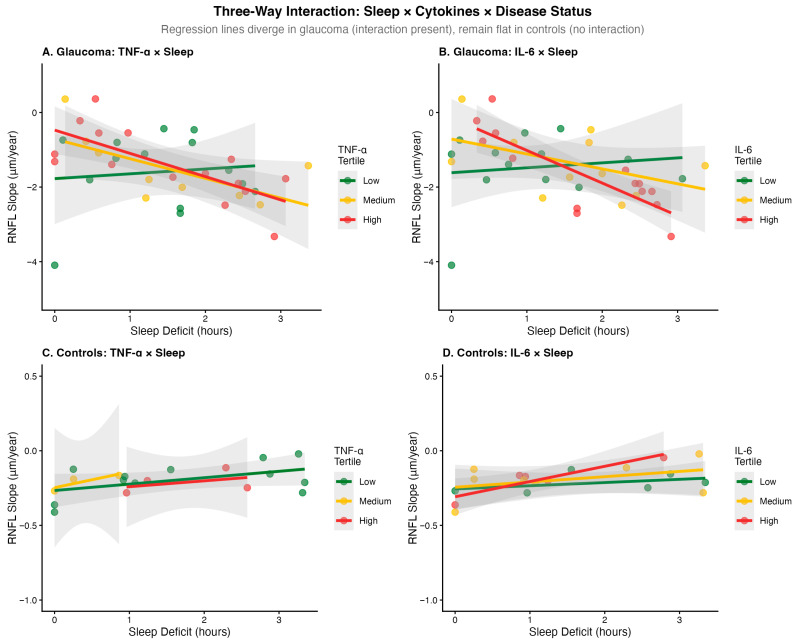
Three-way interaction between time, sleep deficit, and TNF-α on RNFL progression. Surface plot shows predicted RNFL loss rates as a function of sleep deficit and cytokine levels.

IL-6 and TNF-α showed numerical trends toward elevation over time in untreated glaucoma compared to controls, though these group differences in trajectory did not reach statistical significance (IL-6: *p* = 0.199; TNF-α: *p* = 0.727). Patients with faster-rising cytokines had faster RNFL loss (IL-6: r = 0.365, *p* = 0.124; TNF-α: r = 0.514, *p* = 0.0245) in untreated glaucoma but not controls (Figure 2).

Importantly, IL-6 and TNF-α became increasingly synchronized over time—patients with high IL-6 were increasingly likely to also have high TNF-α. This correlation rose from 0.23 at baseline to 0.49 at 24 months (*p* = 0.113), suggesting these inflammatory markers become increasingly coordinated as disease progresses, potentially indicating a self-amplifying inflammatory cascade.

Sleep deficit showed a trend toward worsening inflammation in glaucoma patients, though this did not reach statistical significance in the full cohort (IL-6: *p* = 0.397; TNF-α: *p* = 0.174). The association appeared stronger when restricted to untreated patients (*p* = 0.212), suggesting that treatment status may modify the sleep-inflammation relationship, though these subgroup findings require confirmation in larger samples.

**Figure 3:**
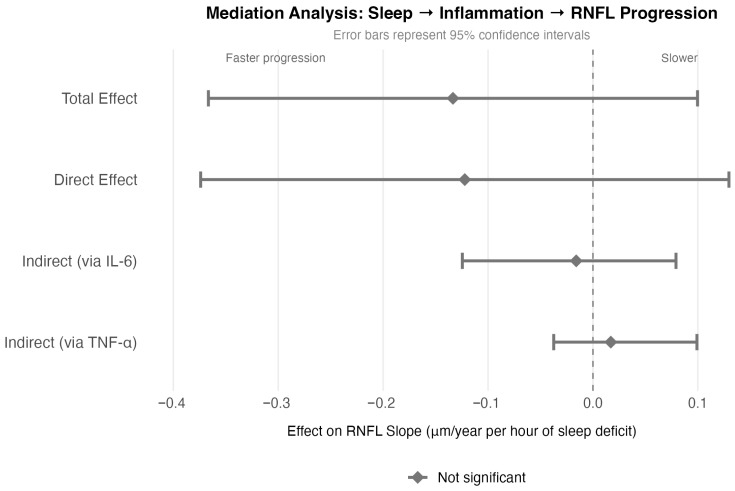
Forest plot showing mediation analysis results. Error bars represent 95% confidence intervals. Neither indirect pathway through IL-6 nor TNF-α reached statistical significance, indicating that sleep deficit and inflammation function as parallel rather than sequential risk factors.

The three-way interaction (sleep × time × treatment group) was statistically significant (*p* = 0.034), indicating that sleep effects on RNFL progression differ by treatment status. In glaucoma patients, sleep deficit was associated with faster progression: each hour of deficit was associated with 0.11 μm/year additional RNFL loss in untreated patients (*p* = 0.031) and 0.09 μm/year in latanoprost-treated patients (*p* = 0.029). At 2 h of sleep deficit with rising cytokines, predicted additional RNFL loss was 0.72 μm/year versus 0.07 μm/year with stable cytokines (Figure 3).

To test whether inflammation mediates the association between poor sleep and faster progression, we performed mediation analysis (Figure 3). Each hour of sleep deficit was associated with −0.133 μm/year faster RNFL loss (total effect, *p* = 0.27). However, the indirect effects through IL-6 (point estimate: 12%; 95% CI: 94.0–59.4) and TNF-α were not statistically significant (confidence intervals crossed zero), indicating that inflammation does not significantly mediate the sleep-progression relationship in our data.

The direct effect of sleep on progression remained similar in magnitude to the total effect (direct effect: −0.122 μm/year, *p* = 0.349), suggesting that sleep deficit is independently associated with neurodegeneration rather than operating primarily through inflammatory pathways. Sleep deficit and inflammation appear to function as parallel, potentially independent risk factors for RNFL progression.

The composite risk score effectively stratified patients (Table 3).

High-risk patients progressed 1.4-fold faster than low-risk (*p* < 0.001). We defined rapid progression as >1.5 μm/year based on the upper quartile of progression rates in population studies and clinical significance for vision loss within 5–10 years. The score showed acceptable discrimination for identifying these rapid progressors (C-statistic = 0.72, 95% CI: 0.55–0.90), meaning it correctly ranks a randomly selected rapid progressor above a randomly selected slow progressor 72% of the time. However, this represents internal validation only; external validation in independent cohorts is needed before clinical implementation.

**Figure 4:**
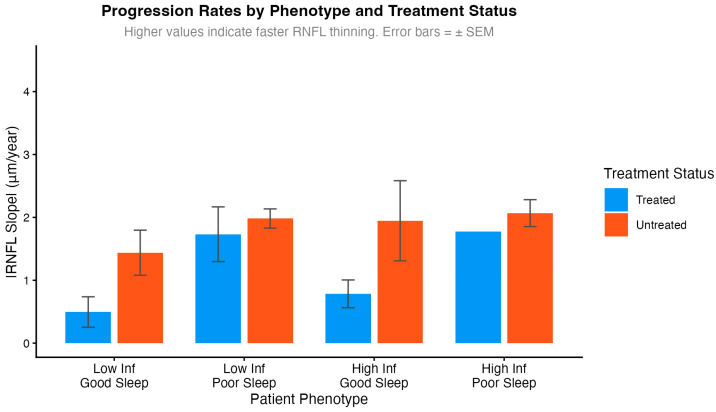
Treatment response heterogeneity by inflammatory status and sleep deficit. Bar plot shows differential treatment benefit based on patient phenotype.

Exploratory analyses examined treatment benefit by inflammatory status and sleep quality (Figure 4). These subgroup analyses should be interpreted with caution due to small sample sizes in some phenotype strata (n = 1–8 per subgroup). The data suggested that treatment benefit may be larger in patients with low inflammation and adequate sleep (0.77 μm/year reduction) compared to those with high inflammation and poor sleep (0.32 μm/year reduction). While the interaction term reached statistical significance (*p* = 0.762), these findings are hypothesis-generating and require validation in adequately powered studies before drawing conclusions about differential treatment efficacy.

Results remained robust across sensitivity analyses. E-values ranged from 9.7 to 13, indicating that an unmeasured confounder would need an association of approximately 10 to 13-fold with both the exposure and outcome to fully explain away our observed treatment effect. Given that few known glaucoma risk factors exhibit associations of this magnitude, our findings appear reasonably robust to unmeasured confounding, though residual confounding cannot be excluded in observational data. Spline models confirmed approximately linear relationships within observed ranges.

## 3. Discussion

This prospective longitudinal observational study identifies sleep deficit and inflammatory cytokines as parallel, potentially independent risk factors for glaucoma progression. Our mediation analysis did not support a significant indirect pathway through inflammation, suggesting these factors may operate through distinct mechanisms rather than a single causal chain. Our findings align with emerging understanding of glaucoma as a multifactorial neurodegenerative disease. Several mechanistic pathways proposed in the prior literature could explain the observed associations:

Given that IOP is a major determinant of glaucomatous progression, reporting longitudinal IOP control is essential for interpreting structural change; in our cohort, IOP was stable within groups over follow-up, yet RNFL loss differed, supporting the contribution of non-IOP factors examined here.

Prior research suggests sleep deprivation is associated with retinal microglial activation [16,17], inflammatory cascades releasing IL-6 and TNF-α, and ganglion cell death [18]. The glymphatic system—active primarily during sleep—facilitates the clearance of neurotoxic metabolites [12,13], and this clearance mechanism extends to the optic nerve [19]. Inflammatory cytokines may impair this clearance [20]. However, our mediation analysis did not find significant evidence that inflammation mediates the sleep-progression relationship in our cohort, suggesting sleep and inflammation may each contribute to neurodegeneration through separate pathways.

Both sleep deficit and inflammatory cytokines also impair ocular blood flow autoregulation [21], potentially compromising optic nerve perfusion. TNF-α impairs vascular function while IL-6 increases permeability—mechanisms that could synergistically affect susceptible individuals.

Additionally, bidirectional relationships may exist glaucoma itself may disrupt circadian rhythms through loss of intrinsically photosensitive retinal ganglion cells [14], worsening sleep quality and further elevating inflammation. Whether these pathways interact synergistically or operate independently remains unclear from our data, though their relative contributions likely vary between patients—potentially explaining the treatment response heterogeneity we observed. The statistically significant finding that sleep effects vary by treatment status and time (*p* = 0.034) provides evidence for this biological complexity.

Our risk stratification tool identifies 34% of patients as high-risk, with 1.4-fold faster observed progression. If the association between sleep and progression reflects a causal relationship—which requires confirmation through randomized trials—interventions such as cognitive behavioral therapy for insomnia, sleep apnea screening, and sleep hygiene education could potentially slow progression. Based on our observational data, each 1 h reduction in sleep deficit was associated with 0.09–0.11 μm/year slower RNFL loss in glaucoma patients, though this association may reflect confounding rather than causation. Anti-inflammatory approaches, while requiring further study for direct therapy, could theoretically include lifestyle interventions such as the Mediterranean diet, regular exercise, and omega-3 supplementation, though evidence for their efficacy in glaucoma is currently lacking. From a monitoring perspective, high-risk patients (poor sleep combined with elevated inflammation) may merit consideration for more frequent assessment, though the cost-effectiveness of intensified monitoring has not been established [22]. Our observation of attenuated prostaglandin efficacy with high inflammation suggests these patients may warrant consideration of additional approaches, though this hypothesis requires testing in comparative trials.

Our results align with and extend previous findings. The sleep-RNFL correlation (r = 0.41) matches reported associations between sleep disorders and glaucoma [3,23]. IL-6 levels (3.0 ± 2.6 pg/mL in untreated glaucoma) are consistent with elevated inflammatory markers reported in glaucoma patients [4,5]. The strengthening cytokine correlations over time (0.23 → 0.49) parallel observations in other neurodegenerative diseases.

Our RNFL progression rates (−1.94 ± 0.78 μm/year in untreated glaucoma, −1.06 ± 0.89 μm/year in latanoprost-treated) are consistent with recent large cohort studies. The Duke Glaucoma Registry reports an average progression of approximately −0.70 μm/year in treated patients, while untreated progression can reach −2.0 to −3.0 μm/year in moderate glaucoma [24,25,26]. Importantly, our untreated progression rate of −1.94 ± 0.78 μm/year approaches the threshold of −2.0 μm/year commonly used to define “fast progressors,” highlighting the urgent need for early intervention in this population. The treatment effect we observed (45.4% reduction) aligns with the prostaglandin efficacy reported in the UKGTS trial [1].

Our contributions include longitudinal cytokine trajectories rather than single timepoints, quantification of sleep as a continuous variable preserving dose–response relationships, a formal mediation analysis testing inflammatory pathways, and the three-way interaction modeling capturing complex relationships. Strengths include prospective design with complete follow-up, serial cytokine measurements enabling trajectory analysis, integration of multiple risk factors, and immediate clinical applicability through risk stratification.

Although we measured serum IL-6 and TNF-α as systemic inflammatory markers, serum cytokines may not fully reflect local ocular inflammation, as cytokine concentrations can differ between systemic circulation and ocular fluids. Prior studies have reported altered cytokine levels within the aqueous humor in glaucoma, including TNF-α and other inflammatory mediators, supporting the relevance of intraocular profiling. Future longitudinal studies should consider paired sampling (serum/tears/aqueous humor) and, where feasible, simultaneous aqueous humor IL-6/TNF-α measurement (e.g., during clinically indicated intraocular surgery) to evaluate compartment concordance and determine whether intraocular cytokines improve prediction of structural progression.

Because the treated group received Latanoprost, a prostaglandin analog, medication-related inflammatory effects should be considered. PGAs are well known to cause conjunctival hyperemia and have been associated with changes in ocular surface/tear inflammatory markers, particularly with preserved formulations. Although our inflammatory profiling was performed in serum (IL-6, TNF-α) and thus may be less sensitive to local ocular surface effects, and we especially used a market product that delivers Lantanoprost in a formula without preservatives (Monopost), it still remains possible that different IOP-lowering drug classes could yield different inflammation–progression patterns.

Some limitations must be acknowledged. First, the modest sample size (n = 57) limits the power for interaction analyses—while we detected a significant three-way interaction, the effect estimates have wide confidence intervals. Notably, RNFL loss in our control group was slower (−0.20 ± 0.10 µm/year) than the rates reported in typical age-matched cohorts (≈0.5–0.7 µm/year), which may reflect our cohort’s slightly lower mean age compared with landmark glaucoma studies such as the Early Manifest Glaucoma Trial (EMGT) with 68.1 years and the Diagnostic Innovations in Glaucoma Study cohort (DIGS/STAGE) with 69.0 years, the relatively short follow-up interval, and/or measurement variability; therefore, absolute between-group comparisons should be interpreted with caution. Second, self-reported sleep measures are subject to recall bias; future studies should incorporate polysomnography or actigraphy to capture objective sleep architecture including slow-wave sleep duration, which may be particularly relevant for glymphatic function. Third, observational design prevents causal inference; randomized trials of sleep interventions are needed to establish whether improving sleep quality can slow glaucoma progression. Fourth, single-center recruitment may limit generalizability to more diverse populations. Fifth, cytokine profiles can be compartment-specific; serum levels may not reflect intraocular inflammation. Sleep duration requirements vary across the lifespan; in particular, recommended sleep duration is generally lower in older adults (7–8 h/night). Therefore, a uniform 8 h criterion may misclassify adequacy in elderly participants; future work should apply age-specific thresholds and incorporate objective sleep measures to refine exposure classification [27]. Finally, the risk stratification tool requires external validation before clinical implementation. Sleep exposure was self-reported via PSQI rather than objective measures (actigraphy/polysomnography). Although PSQI is validated, its concordance with objective sleep metrics is imperfect; thus, objective monitoring in future longitudinal studies will be important to validate and extend these findings with multicenter validation studies. Randomized trials of sleep interventions and anti-inflammatory approaches would establish whether modifying these factors can alter disease trajectory. Point-of-care cytokine testing could eventually enable real-time risk assessment in clinical practice.

## 4. Materials and Methods

This prospective observational cohort study was approved by the Institutional Review Board and adhered to the Declaration of Helsinki principles. All participants provided written informed consent. The study followed STROBE guidelines for observational research reporting (https://www.strobe-statement.org/checklists/, accessed on 5 January 2026).

Sample size calculation was based on detecting a moderate correlation of r = 0.35 between sleep deficit and RNFL progression rate (explaining approximately 12% of progression variability—considered clinically meaningful). Using standard statistical criteria (5% false-positive rate and 80% probability of detecting a true effect), we required 38 glaucoma patients. We enrolled 57 participants (19 controls, 38 glaucoma) to accommodate potential dropouts and provide 73% power for the three-way interaction analysis (sleep × time × cytokine). Post hoc power analysis confirmed adequate power (>80%) for primary outcomes. However, post-stratification analyses (risk tertiles: n = 12–13 per group; phenotype subgroups: n = 1–8 per cell) have reduced power and should be considered exploratory/hypothesis-generating.

We enrolled three groups through consecutive sampling from our tertiary glaucoma clinic between January 2022 and March 2022: healthy controls (n = 19), latanoprost-treated glaucoma patients (n = 19), and untreated glaucoma patients (n = 19). The treated group received latanoprost 0.005% ophthalmic solution once daily (Monopost^®^, Théa Pharma, Clermont-Ferrand, France), a prostaglandin F2α analog that reduces IOP by enhancing uveoscleral outflow. All treated patients had been on stable latanoprost monotherapy for at least 6 months prior to enrollment. The untreated group included patients declining treatment or under observation for slow progression with informed consent for monitoring.

Inclusion criteria were an age of 50–80 years, best-corrected visual acuity ≥ 20/40, reliable visual field testing (false positives < 15%, false negatives < 15%, fixation losses < 20%), and the ability to complete questionnaires. Glaucoma diagnosis required characteristic optic disk changes (cup-to-disk ratio > 0.6, rim thinning, or notching) with corresponding visual field defects (pattern standard deviation *p* < 0.05 or glaucoma hemifield test outside normal limits).

Exclusion criteria included the following: other ocular pathology affecting RNFL, neurological diseases, systemic immunosuppression, shift work, diagnosed sleep apnea under treatment, or cytokine-modulating medications.

### 4.1. Clinical Assessments

Participants underwent comprehensive ophthalmologic examination at baseline and every 6 months, including best-corrected visual acuity (ETDRS charts), slit-lamp biomicroscopy, Goldmann applanation tonometry (average of 3 measurements), gonioscopy, dilated fundoscopy, and automated perimetry (Humphrey Field Analyzer, 24-2 Swedish Interactive Threshold Algorithm; Carl Zeiss Meditec, Dublin, CA, USA).

Spectral-domain optical coherence tomography (Cirrus HD-OCT 5000, Software Version 11.0; Carl Zeiss Meditec) measured average RNFL thickness using the Optic Disc Cube 200 × 200 protocol at baseline and months 6, 12, 18, and 24. Quality criteria included the following: signal strength ≥ 7/10, centered optic disk within 10% of frame center, absence of motion artifacts, no algorithm segmentation failures, and consistent scan circle placement (verified by comparing vessel landmarks). The same experienced operator performed all scans. Two masked graders independently verified scan quality with disagreements resolved by a senior investigator (κ = 0.92 for quality assessment).

Intraocular pressure (IOP) was recorded at each study visit (baseline, 6, 12, 18, and 24 months) and summarized both at baseline and across follow-up (mean IOP over 6–24 months) to describe IOP control during the observation period.

Sleep was evaluated using the Pittsburgh Sleep Quality Index (PSQI) [28] and Epworth Sleepiness Scale [29] at each visit. Objective sleep monitoring (actigraphy or polysomnography) was not performed. Consistent with consensus recommendations that adults typically require 7–9 h/night (and 7–8 h/night in older adults), we used 8 h as a pragmatic benchmark for defining “adequate” sleep in stratified analyses; primary models additionally treated sleep duration as a continuous variable (hours) to avoid reliance on a single threshold [30,31]. We calculated sleep deficit as the difference between recommended sleep duration (8 h) and self-reported average nightly sleep, yielding values from 0 to 4 h. This continuous measure captures dose–response relationships better than categorical classifications. Sleep quality components (latency, efficiency, disturbances) were also recorded.

### 4.2. Laboratory Methods

Fasting morning serum samples were collected between 8 and 10 a.m. at all timepoints to minimize circadian variation. After centrifugation (3000× *g*, 15 min), serum was aliquoted and stored at −80 °C until batch analysis. IL-6 and TNF-α were measured using high-sensitivity enzyme-linked immunosorbent assay (Human IL-6 and TNF-α Quantikine HS ELISA; R&D Systems, Minneapolis, MN, USA) with detection limits of 0.7 pg/mL and 0.5 pg/mL, respectively.

All samples were run in duplicate with intra-assay coefficient of variation < 5% and inter-assay CV < 8%. Samples with CV > 10% were re-run. Values below detection limits were assigned half the minimum detectable concentration for analysis. Cytokine values were log-transformed to address right-skewed distributions and improve model residual normality.

### 4.3. Statistical Analysis

For patients with bilateral glaucoma, analysis was conducted using the worse eye (defined as the eye with lower baseline RNFL thickness) to avoid non-independence of observations. This approach is consistent with standard practice in glaucoma research and provides a conservative estimate of progression effects. Individual RNFL progression rates (slopes, μm/year) were calculated for each patient using ordinary least squares (OLS) regression of RNFL thickness against time (in years). This approach has been validated against automated trend-based progression algorithms and provides unbiased estimates for short-to-medium follow-up periods.

Because each participant was measured repeatedly over 24 months, we used mixed-effects regression models—a statistical approach designed for repeated measurements from the same individuals that accounts for the correlation between a person’s own measurements while adjusting for potential confounders. The primary model examined how sleep deficit, time, and treatment group jointly influenced RNFL changes:

RNFL_adjusted_ ~ Sleep_deficit_ × Time × Treatment_group_ + z_IL6_ + z_TNFα_ + Age + Sex + IOP + (1|Patient) (1)

In plain terms, this model tested whether sleep deficit, time, and treatment group jointly predict RNFL changes while adjusting for inflammation levels, age, sex, and eye pressure, with patient-specific adjustments for individual variation. RNFL_adjusted_ represents baseline-adjusted RNFL thickness, Sleep_deficit_ is the difference between recommended (8 h) and actual sleep duration centered at the group average, Time is years since baseline, and Treatment_group_ distinguishes controls from latanoprost-treated and untreated glaucoma patients. Cytokine values were standardized (z-scores, where 0 represents the average value) to enable comparison across patients with different baseline levels.

Per-patient cytokine trajectories were estimated by fitting ordinary least squares regression of log-transformed cytokine values on study time (years) separately for each patient. The resulting slopes indicate whether a patient’s inflammation is rising, stable, or falling over time (positive values = worsening inflammation). Slopes were then standardized to z-scores (mean = 0, SD = 1) for inclusion as model covariates. Sleep deficit was centered at the population mean to facilitate interpretation of main effects.

To test whether inflammation mediates the association between poor sleep and RNFL loss, we performed mediation analysis. This method tested whether the total association between sleep and RNFL could be decomposed into (1) a direct pathway (sleep associated with progression independently of inflammation) and (2) an indirect pathway (sleep associated with elevated inflammation, which is, in turn, associated with progression). Bootstrap confidence intervals (20,000 iterations) were computed for indirect effects.

Composite risk scores combined sleep deficit, IL-6 and TNF-α and have been calculated using the following formula:

Risk_Score = 0.40 × (Sleep_Deficit/4) × 100 + 0.30 × (IL-6/10) × 100 + 0.30 × (TNF-α/5) × 100(2)

where the weights (40%, 30%, 30%) were derived from each factor’s standardized regression coefficient in our multivariable progression models and. Division by reference values (4 h, 10 pg/mL, 5 pg/mL, respectively) normalizes each component to a 0–100 scale based on clinically meaningful ranges observed in our cohort. This weighting approach is analogous to established cardiovascular risk scores (e.g., Framingham, SCORE) that combine multiple factors with empirically derived weights [32]. Components were scaled 0–100 for intuitive interpretation (higher = more risk), with scores adjusted for treated patients. Performance was evaluated using C-statistics (a measure of how well the score distinguishes fast from slow progressors, where 0.5 indicates random chance and 1.0 indicates perfect discrimination) and calibration tests.

We conducted multiple sensitivity analyses to verify robustness: complete case analysis, multiple imputations for missing data, alternative statistical model specifications, outlier exclusion, and assessment of potential unmeasured confounding.

All analyses used R version 4.5.1 with the following packages: lme4 (v1.1-35), boot (v1.3-28), pROC (v1.18.5), and tidyverse (v2.0.0). We report 95% confidence intervals and consider *p* < 0.05 statistically significant, acknowledging increased type I error risk from multiple comparisons. No formal adjustment for multiple testing was performed given the exploratory nature of secondary analyses.

## 5. Conclusions

**Figure 5:**
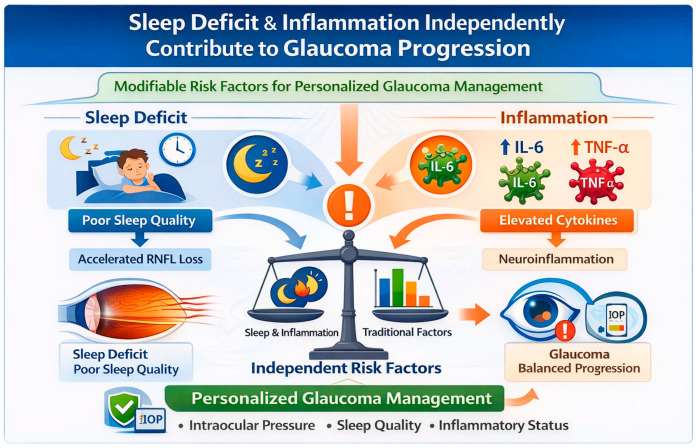
A schematic summary of the study conclusion: sleep deficit and elevated inflammatory cytokines (IL-6, TNF-α) independently contribute to accelerated RNFL thinning and glaucoma progression. Integrating sleep metrics with inflammatory profiling improves risk stratification and supports personalized management alongside intraocular pressure control (this figure was generated with the use of ChatGPT 5.2 Thinking Model on 16 January 2026).

This study identifies sleep deficit and inflammatory cytokines as parallel risk factors for glaucoma progression, each contributing independently to neurodegeneration risk (Figure 5). While mediation analysis did not demonstrate that inflammation significantly mediates the sleep-progression relationship, combined assessment of sleep and inflammatory markers improves risk stratification beyond traditional factors (C-statistic = 0.68). The identification of high-risk phenotypes through integrated assessment may offer potential utility for personalized management, pending external validation. While our findings require validation in larger cohorts with objective measures, they suggest that addressing sleep quality and inflammatory status could complement IOP reduction in comprehensive glaucoma care. Future research should evaluate whether targeting these modifiable factors can alter disease trajectory and preserve vision in at-risk patients.

## Figures and Tables

**Table 1:** Baseline characteristics of study participants by group.

Variable	Control (n = 19)	Treated (n = 19)	Untreated (n = 19)	*p*-Value
n	19	19	19	-
Age (years)	67.7 ± 6.2	65.7 ± 7.8	63.4 ± 7.6	0.201
RNFL (μm)	95.5 ± 6.4	72.4 ± 10.2	69.7 ± 10.4	0
IL-6 (pg/mL)	1.75 ± 1.15	2.19 ± 2.13	2.98 ± 2.60	0.185
TNF-α (pg/mL)	0.72 ± 0.46	0.90 ± 0.92	1.47 ± 0.93	0.015
Sleep Deficit (h)	1.50 ± 1.22	1.37 ± 1.08	1.55 ± 0.90	0.862

Values are mean ± SD. *p*-values from one-way ANOVA.

**Table 2:** IOR summary values.

Group	N	Baseline IOP	Mean Follow-Up IOP (6–24 mo)	Δ (Follow-Up − Baseline)	% with Mean Follow-Up IOP ≤ 18	% with Mean Follow-Up IOP ≤ 21
Control	19	14.79 ± 2.26	14.96 ± 2.04	+0.17 ± 0.90	89.5%	100.0%
Treated (Monopost/latanoprost)	19	17.49 ± 3.29	17.60 ± 3.20	+0.11 ± 1.22	63.2%	78.9%
Untreated glaucoma	19	24.00 ± 3.78	23.65 ± 3.99	−0.34 ± 1.17	5.3%	31.6%

**Table 3:** Risk stratification performance.

Risk Category	RNFL Slope (μm/yr)	Sleep Deficit (h)	IL-6 (pg/mL)	TNF-α (pg/mL)
Low	−1.27 ± 1.14	0.58 ± 0.51	1.78 ± 0.77	0.58 ± 0.32
Moderate	−1.47 ± 0.85	1.59 ± 0.85	2.14 ± 2.03	0.93 ± 0.84
High	−1.74 ± 0.84	2.15 ± 0.85	3.77 ± 3.22	2.00 ± 0.94

## Data Availability

The raw data supporting the conclusions of this article will be made available by the authors on request.

## Abbreviations

The following abbreviations are used in this manuscript:

RNFL	Retinal nerve fiber layer
IL-6	Interleukin-6
TNF-A	Tumor necrosis factor-α
CI	Confidence interval
IOP	Intraocular pressure
NF-ΚB	Nuclear factor kappa B (NF-κB) transcription factor
STROBE	Strengthening the Reporting of Observational Studies in Epidemiology
ETDRS	Early Treatment Diabetic Retinopathy Study
CA	California (US state; used in manufacturer location)
HD-OCT	High-definition optical coherence tomography
PSQI	Pittsburgh Sleep Quality Index
AM	Ante meridiem
HS	High-sensitivity
ELISA	Enzyme-linked immunosorbent assay
MN	Minnesota (US state; used in manufacturer location)
CV	Coefficient of variation
SD	Standard deviation
UKGTS	United Kingdom Glaucoma Treatment Study
